# New destination vectors facilitate Modular Cloning for Chlamydomonas

**DOI:** 10.1007/s00294-022-01239-x

**Published:** 2022-04-16

**Authors:** Justus Niemeyer, Michael Schroda

**Affiliations:** Molecular Biotechnology and Systems Biology, TU Kaiserslautern, Paul-Ehrlich-Straße 23, 67663 Kaiserslautern, Germany

**Keywords:** Synthetic Biology, Antibiotic resistance, Golden Gate cloning, Microalgae, *Chlamydomonas reinhardtii*

## Abstract

Synthetic Biology is revolutionizing biological research by introducing principles of mechanical engineering, including the standardization of genetic parts and standardized part assembly routes. Both are realized in the Modular Cloning (MoClo) strategy. MoClo allows for the rapid and robust assembly of individual genes and multigene clusters, enabling iterative cycles of gene design, construction, testing, and learning in short time. This is particularly true if generation times of target organisms are short, as is the case for the unicellular green alga *Chlamydomonas reinhardtii*. Testing a gene of interest in Chlamydomonas with MoClo requires two assembly steps, one for the gene of interest itself and another to combine it with a selection marker. To reduce this to a single assembly step, we constructed five new destination vectors. They contain genes conferring resistance to commonly used antibiotics in Chlamydomonas and a site for the direct assembly of basic genetic parts. The vectors employ red/white color selection and, therefore, do not require costly compounds like X-gal and IPTG. mCherry expression is used to demonstrate the functionality of these vectors.

## Introduction

**Fig. 1:**
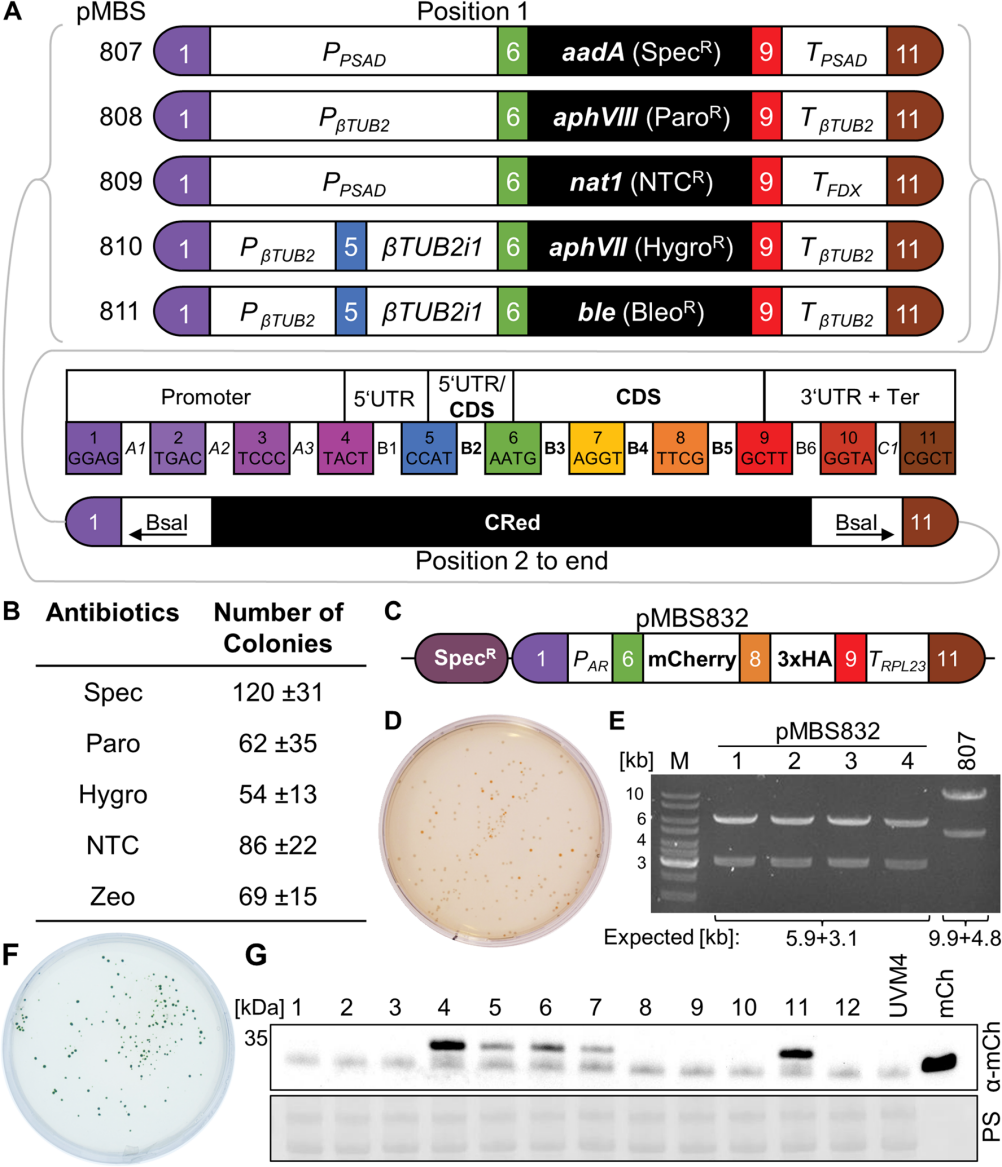
Level 2 vector design and proof of functionality in *E. coli* and Chlamydomonas. **A** Genetic parts used to assemble transcription units in position 1, mediating resistance to spectinomycin (Spec^R^), paromomycin (Paro^R^), nourseothricin (NTC^R^), hygromycin (Hygro^R^), and zeocin (Bleo^R^). Colored boxes represent MoClo fusion sites with the respective 4-nt junctions. Position 2 to end contains the CRed operon for red/white color selection flanked by BsaI sites for the replacement of CRed by directionally assembling level 0 parts. **B** Average number of transformant colonies obtained upon transformation of Chlamydomonas with the five vectors and selection on agar plates containing the indicated antibiotic concentrations (*n* = 5–6 ± SD). **C** Assembly of level 0 genetic parts into position 2 (to end) of pMBS807. *P*_AR_: *HSP70A-RBCS2* promoter. **D** Transformation of *E. coli* with the MoClo restriction/ligation reaction for the assembly of level 0 parts into pMBS807 (yielding pMBS832) and red/white color selection on an LB-agar plate containing kanamycin. **E** Restriction analysis of plasmids prepared from four white *E. coli* transformants and of empty pMBS807 using restriction enzyme NdeI. **F** Transformation of Chlamydomonas UVM4 with pMBS832 and selection on TAP-agar plates containing spectinomycin. **G** Immunoblot analysis of total proteins from 12 randomly picked, spectinomycin-resistant transformants generated with pMBS832 and untransformed UVM4. 15 ng recombinant mCherry (mCh) were loaded as positive control. *PS* ponceau staining

Classical cloning strategies based on type II restriction enzymes have a number of disadvantages: typically, only two fragments can be combined at a time and the assembly reaction is not very efficient. Due to the use of a broad variety of restriction enzymes, genetic elements are not standardized and, thus, hardly interchangeable. Synthetic Biology, in contrast, employs principles of mechanical engineering, including the standardization of basic genetic parts and standardized part assembly routes. Both principles are realized in the Modular Cloning (MoClo) system (Weber et al. 2011). Here, assembly is achieved by Golden Gate cloning using the type IIS restriction enzymes BsaI and BpiI and T4 DNA ligase, allowing the efficient assembly of multiple genetic parts in a single reaction. Standardization of the parts means that they must lack BpiI and BsaI recognition sites and are cloned into specific level 0 destination vectors. BsaI digestion of these vectors releases the parts with characteristic 4-nt overhangs corresponding to defined fusion sites separating the functional parts of a transcription unit (promoter, 5′-UTR, signal peptide, CDS, tag, 3′-UTR, terminator) (Patron et al. 2015; Weber et al. 2011) (Fig. 1A). These parts are then directionally assembled into a transcription unit within a level 1 destination vector present in the restriction/ligation reaction. Selection of correctly assembled level 1 constructs is realized by *lacZ*-based blue/white color selection, requiring the costly compounds X-gal and IPTG. Several transcription units in level 1 vectors can then be released by cleavage with BpiI and assembled into a level 2 destination vector present in the same reaction. Here, the selection of correct assemblies relies on red/white color selection based on an artificial bacterial operon responsible for canthaxanthin biosynthesis, not requiring costly compounds (Weber et al. 2011). This second assembly step allows the construction of multigene clusters, e.g., encoding the enzymes responsible for a complete metabolic pathway.

Efficient eukaryotic transgene expression depends on strong promoters, enhancers, and terminators, a proper codon usage, and often on the presence of suitable introns [reviewed in Schroda (2019)]. These limitations require organism-specific part collections for a MoClo-based Synthetic Biology strategy. Accordingly, a MoClo toolkit with 119 standardized parts has been established previously for the green alga *Chlamydomonas reinhardtii* (Crozet et al. 2018), and this kit is rapidly expanding (de Carpentier et al. 2020; Dementyeva et al. 2021; Einhaus et al. 2021; Geisler et al. 2021; Kiefer et al. 2021; Mehrshahi et al. 2020; Niemeyer et al. 2021).

Testing the activity of a single gene probably is the most frequent goal of transgenic approaches in Chlamydomonas, e.g., if mutants derived from the CLiP collection are to be complemented (Li et al. 2016; Spaniol et al. 2022; Theis et al. 2020). If this is done with the MoClo system, a level 1 transcription unit containing the gene of interest is assembled first. The latter is then assembled together with a level 1 transcription unit containing a resistance marker into a level 2 destination vector. Although the assembly is fast and robust, two cloning steps for the analysis of a single gene appears inappropriate in terms of time and resources required.

In this report we have addressed this problem and provide five new level 2 vectors already containing markers conferring resistance against commonly used antibiotics, into which level 0 genetic parts can directly be assembled. This reduces the time and resources required by half. Since the selection for a successful assembly is based on red/white instead of blue/white color selection, the required resources are further reduced.

## Methods

### Strains and culture conditions

For the transformation of *E. coli*, TOP10 cells (Invitrogen) were made competent with CaCl_2_ and transformed via the heat shock method (Sambrook et al. 1989). Bacteria were selected at 37 °C on LB-agar plates (1.5% w/v) containing 50 µg/mL kanamycin. For the transformation of *Chlamydomonas reinhardtii*, strain UVM4 (Neupert et al. 2020) was grown in Tris–Acetate–Phosphate (TAP) medium (Kropat et al. 2011) on a rotatory shaker at a constant light intensity of  ~ 40 µmol photons m^–2^ s^–1^. Transformation was done via the glass bead method (Kindle 1990) using 5 × 10^7^ UVM4 cells and 1 µg of DNA linearized with NotI (NEB). Cells were plated onto TAP plates containing 1.5% w/v agar and either spectinomycin (Sigma, 100 µg/mL), paromomycin (Sigma, 10 µg/mL), hygromycin B (Roth, 15 µg/mL), zeocin (Invitrogen, 0.5 µg/mL) or nourseothricin (Sigma, 10 µg/mL) and incubated in dim light until colonies became visible.

### Cloning

For conferring resistance of Chlamydomonas transformants to various antibiotics, level 1 transcription units (pMBS) were assembled using level 1 destination vector pICH47732 (forward position 1) (Weber et al. 2011) and the following level 0 parts from the Chlamydomonas MoClo kit (pCM) (Crozet et al. 2018) or generated by others (references): resistance to nourseothricin (pMBS804): *PSAD* promoter + 5′-UTR_A1-B2 (pCM0-016), *nat1*-CDS_B3-5 (MoClo-adapted construct provided by Thomas Baier), and *FDX* terminator_B6-C1 (Einhaus et al. 2021). Resistance to hygromycin (pMBS805): *βTUB2* promoter + 5′-UTR_A1-B1 (pCM0-013), *βTUB2*i1 Intron_B2 (pCM0-041), *aphVII*-CDS_B3-B5 (pCM0-073), and *βTUB2* terminator_B6-C1 (pCM0-118). Resistance to zeocin (pMBS806): *βTUB2* promoter + 5′-UTR_A1-B1 (pCM0-013), *βTUB2*i1 Intron_B2 (pCM0-041), *bleoRi*-CDS_B3-B5 (pCM0-077), and *βTUB2* terminator_B6-C1 (pCM0-118). Resistance to paromomycin (pMBS503): *βTUB2* promoter + 5′-UTR_A1-B2 (pCM0-021), *aphVIII*-CDS_B3-B5 (pCM0-074), and *βTUB2* terminator_B6-C1 (pCM0-118). For resistance to spectinomycin, level 1 module pCM1-01 containing the *aadA* gene flanked by *PSAD* promoter and terminator was used.

To equip our new level 2 destination vectors with red/white color selection, level 2 destination vector pAGM4673 was used as template to amplify the CRed operon by PCR. Q5 polymerase (NEB) was employed following the manufacturer's instructions. The oligonucleotides 5′-TTGAAGACAA*GCAAGGAG*TGAGACCCAGTGGTATGGGGTACCGCACG-3′ and 5′-TTGAAGACAA*TCCCAGCG*TGAGACCCACTTGAGTGGTTTTTAATAAAAAAGCCCCG-3′ introduced BsaI recognition sites (underlined) immediately flanking the CRed operon, giving rise to *GGAG* (fusion site 1) and *CGCT* (fusion site 11) overhangs upon BsaI digestion, with BsaI recognition sites remaining on the CRed fragment. Furthermore, the oligonucleotides introduced BbsI recognition sites (underlined) peripheral to the BsaI sites, giving rise to *GCAA* and *GGGA* overhangs upon BbsI digestion, with BbsI sites being removed from the CRed fragment. The five new level 2 destination vectors (pMBS807-811) were assembled by combining the purified PCR product containing CRed (forward position 2 to end), one of the five level 1 resistance constructs (forward position 1), and the level 2 destination vector pAGM4673 via digestion with BbsI (NEB) and ligation with T4 DNA ligase (NEB) at 37 °C for 5 h, as described previously (Crozet et al. 2018). After transformation of *E. coli* TOP10 with the assembled construct, red colonies were picked for plasmid preparation using the NucleoSpin Plasmid EasyPure kit (Macherey-Nagel) according to the manufacturer’s instructions. The introduced modifications were confirmed by Sanger sequencing. The new level 2 destination vectors (pMBS807-811) were added to the Chlamydomonas MoClo toolkit and can be ordered from the Chlamydomonas Resource Center (www.chlamycollection.org).

The newly constructed destination vector pMBS807, conferring resistance to spectinomycin, was assembled with level 0 parts pCM0-017 (*HSP70A-RBCS2* promoter + 5′-UTR_A1-B2), pCM0-067 (*mCherry(i1)*_B3-B4), pCM0-100 (*3xHA*_B5), and pCM0-119 (*RPL23* terminator_B6-C1) by digestion with BsaI (NEB) and ligation with T4 DNA ligase, giving rise to pMBS834.

### Protein analysis

Cells were pelleted and resuspended in 50 mM DTT, 50 mM Na_2_CO_3_, 2.5% (w/v) SDS and 15% (w/v) sucrose, boiled for 2 min at 95 °C and centrifuged. After determination of the chlorophyll content (Porra et al. 1989), a sample volume corresponding to 1 µg chlorophyll was subjected to SDS-PAGE (10% acrylamide). After semidry blotting, immunodetection was performed by enhanced chemiluminescence using the INTAS imaging system as described previously (Hammel et al. 2020). The antiserum against mCherry (1:5000) was kindly provided by Felix Willmund, the secondary antibody was anti-rabbit-HRP (Sigma-Aldrich, 1:10,000). Recombinant HA-tagged mCherry was prepared as reported previously (Kiefer et al. 2021).

## Results and discussion

**Fig. 2:**
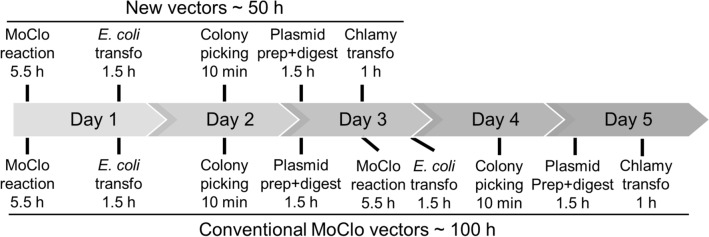
Time elapsing between construct assembly and Chlamydomonas transformation with the new level 2 destination vectors compared to the conventional ones

We first assembled five level 1 transcription units with bacterial genes that have previously been shown to confer robust antibiotic resistance to Chlamydomonas if expressed as nuclear transgenes and are routinely used for genetic engineering of Chlamydomonas. These genes are *aadA* (spectinomycin resistance) (Meslet-Cladiere and Vallon 2011), *aphVIII* (paromomycin resistance) (Sizova et al. 2001), *nat1* (nourseothricin resistance) (Yang et al. 2019), *aphVII* (hygromycin resistance) (Berthold et al. 2002), and *ble* (phleomycin resistance) (Stevens et al. 1996). To avoid the double use of the same promoter/terminator for driving gene of interest and resistance gene expression, we equipped the resistance gene with the *PSAD* or *βTUB2* promoter and different combinations of terminators, including *PSAD*, *FDX* or *βTUB2* (Fig. 1A). The five level 1 resistance cassettes were then inserted into the first position of a level 2 destination vector, and the second to end position was occupied by the CRed operon flanked by BsaI sites (Fig. 1A). One of the resulting five vectors can now be incubated with level 0 plasmids containing the gene of interest and genetic parts of choice. Upon digestion with BsaI and ligation with T4 DNA ligase, all parts are directionally assembled into the site previously occupied by CRed, thereby allowing red/white color selection (Weber et al. 2011). We first tested the functionality of the resistance markers in the five new destination vectors by transforming the empty vectors into Chlamydomonas and plating transformed cells on the respective antibiotic. As shown in Fig. 1B, we routinely obtained between 50 and 120 colonies per plate, i.e., a sufficiently large number for further screening. Next, we exemplarily tested the vector conferring spectinomycin resistance for its functionality regarding the directional assembly of level 0 genetic parts into position 2. The parts chosen were the *HSP70A-RBCS2* promoter, sequences encoding mCherry and a 3xHA tag, and the *RPL23* terminator, all from the Chlamydomonas MoClo kit (Crozet et al. 2018) (Fig. 1C). 86% of the 160 *E. coli* transformants generated with the MoClo restriction/ligation reaction were white, indicating functionality of the red/white color selection (Fig. 1D). Restriction digestion of plasmids prepared from four randomly picked white transformants gave the expected fragment sizes for all of them, indicating that the assembly works with high efficiency (Fig. 1E). Transformation of one of the four linearized plasmids into Chlamydomonas and selection on spectinomycin resulted in more than 100 transformants per plate (Fig. 1F). Twelve of them were randomly picked and total cell proteins analyzed for the accumulation of mCherry-3xHA by immunoblotting. As shown in Fig. 1G, mCherry accumulated to detectable levels in five transformants with strong expression detected in two. This result is typically obtained with conventional level 2 assemblies in the UVM4 strain for expressing roGFP or SBPase (Hammel et al. 2020; Niemeyer et al. 2021), and thus provides the proof of the functionality of the new target vectors. With these vectors, the time elapsing between construct assembly and transformation into Chlamydomonas can be reduced from 5 days (~ 100 h) to 3 days (~ 50 h) (Fig. 2).

A frequent application in Chlamydomonas research is the complementation of mutants that have been generated at large scale via insertional mutagenesis with cassettes conferring resistance against antibiotics like zeocin or paromomycin (Li et al. 2016; Wakao et al. 2021). Often a selection on the complementing gene is not possible and in this case a different selection marker than the one used for insertional mutagenesis is required. For such cases, the five selection markers implemented in our new vectors offer sufficient choice.
